# A Case of Oral Glomeruloid Hemangioma Without Systemic Conditions

**DOI:** 10.7759/cureus.21705

**Published:** 2022-01-29

**Authors:** Rita R Roy, Katsumitsu Shimada, Hiromasa Hasegawa

**Affiliations:** 1 Hard Tissue Pathology Unit, Graduate School of Oral Medicine, Matsumoto Dental University, Shiojiri, JPN; 2 Department of Oral Pathology, Matsumoto Dental University, Shiojiri, JPN

**Keywords:** nestin, cd146, stromal cell, glomeruloid hemangioma, oral cavity

## Abstract

Glomeruloid hemangioma is a rare variant of hemangioma that is accompanied by polyneuropathy, organomegaly, endocrinopathy, monoclonal gammopathy, and skin abnormalities (POEMS) syndrome and, rarely, by thrombocytopenia, anasarca, fever, reticulin fibrosis, and organomegaly (TAFRO) syndrome. This report presents the case of a 78-year-old male who presented with a hemorrhagic nodule on the tongue without any other systemic diseases. Microscopically, the lesion was a lobular proliferation extending from the lamina propria to muscular tissue. Some intravascular nodules with irregular vascular lumens closely resembled renal glomeruli. Each nodule consisted of plump endothelial and stromal cells that partially showed vacuolated cytoplasm containing eosinophilic and periodic acid-Schiff (PAS)-positive globules. Immunohistochemically, IgG-positive deposition was noted within CD31-positive cells. Many plump stromal cells were positive for CD31, CD146, nestin, and type IV collagen but not α-smooth muscle actin (αSMA). These results reflect the proliferation of immature endothelial cells and pericytes, which might characterize this unique lesion. Microscopically, this case revealed glomeruloid hemangioma without systemic conditions related to POEMS, and composed of an intravascular proliferation of immature endothelial and pericytic stromal cells.

## Introduction

The term "glomeruloid hemangioma" (GH) was first coined by Chan et al. in 1990 as a histologically distinctive cutaneous hemangioma that showed capillaries aggregating within ectatic vascular spaces reminiscent of renal glomeruli [1]. This unique variety of benign vascular lesion affects several sites including the breast, abdominal wall, chest wall, shoulder, upper arm, and face but oral cases have not been reported yet [1,2].

GH is a manifestation of polyneuropathy, organomegaly, endocrinopathy, M protein, and skin abnormalities (POEMS) syndrome and can be accompanied by Castleman disease or thrombocytopenia, anasarca, fever, reticulin fibrosis, and organomegaly (TAFRO) syndrome [3]. It is important to identify GH in routine histopathological diagnosis because it is considered a specific marker of POEMS syndrome [2]. Initially, all GHs were thought to be accompanied by POEMS syndrome, but there are a few reported cases in which GH in skin was not accompanied without POEMS syndrome [4]. Some cases developed POEMS syndrome 6-10 years after the first GH onset [5,6].

Here, we report a case of vascular lesion showing a typical histological feature of GH in dorsal surface of the tongue but it lacks any systemic abnormality related to POEMS syndrome at the time of the diagnosis. As the diagnosis of GH without POEMS syndrome is challenging, several differential diagnoses including lobular capillary hemangioma and papillary hemangioma lobular capillary hemangioma are discussed in this report.

## Case presentation

**Figure 1:**
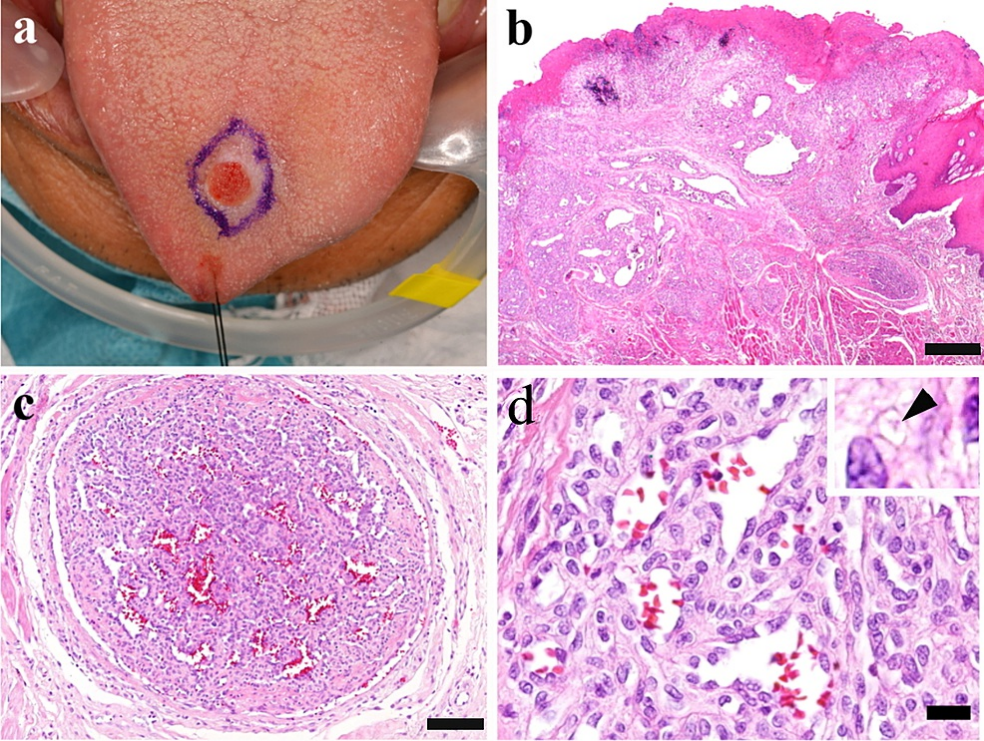
Macroscopic and histopathological features: (a) A slightly elevated and ulcerated lesion of the tongue; (b) A lobular proliferation extends from the mucosa to muscular tissue; (c) Some nodules mimic renal glomeruli; (d) Bland tumor cells show cytoplasmic vacuole containing eosinophilic globules (inset: arrowhead) Scale bars: 1.0 mm in (b), 100 µm in (c), and 20 µm in (d)

A 78-year-old male patient noted a nodule on his tongue with repeated hemorrhage two weeks before visiting a hospital. Although he had been hospitalized for cerebral infarction twice in the last year, he had no other known systemic lesions. On examination, a slightly elevated lesion with ulceration was found in the dorsal surface of the mid-anterior tongue (Figure 1a), and no other mucosal lesions were noted. The whole excised lesion measured 12 × 10 mm. Microscopically, ulcerated mucosa was covered with granulation tissue and was accompanied with fibrin exudation. The lesion was a lobular proliferation with various sizes of dilated vascular spaces, extending from lamina propria to muscle layers mimicking invasion (Figure 1b). Some intravascular nodules comprising clusters of capillaries closely resembled renal glomeruli, accompanied with papillary projection of endothelial cells into vascular lumens in parts (Figure 1c). Irregular vascular channels were lined by plump endothelium cells, and their interstitial spaces were occupied by plump stromal cells. Intracytoplasmic vacuoles of bland tumor cells contained eosinophilic globules (Figure 1d).

**Figure 2:**
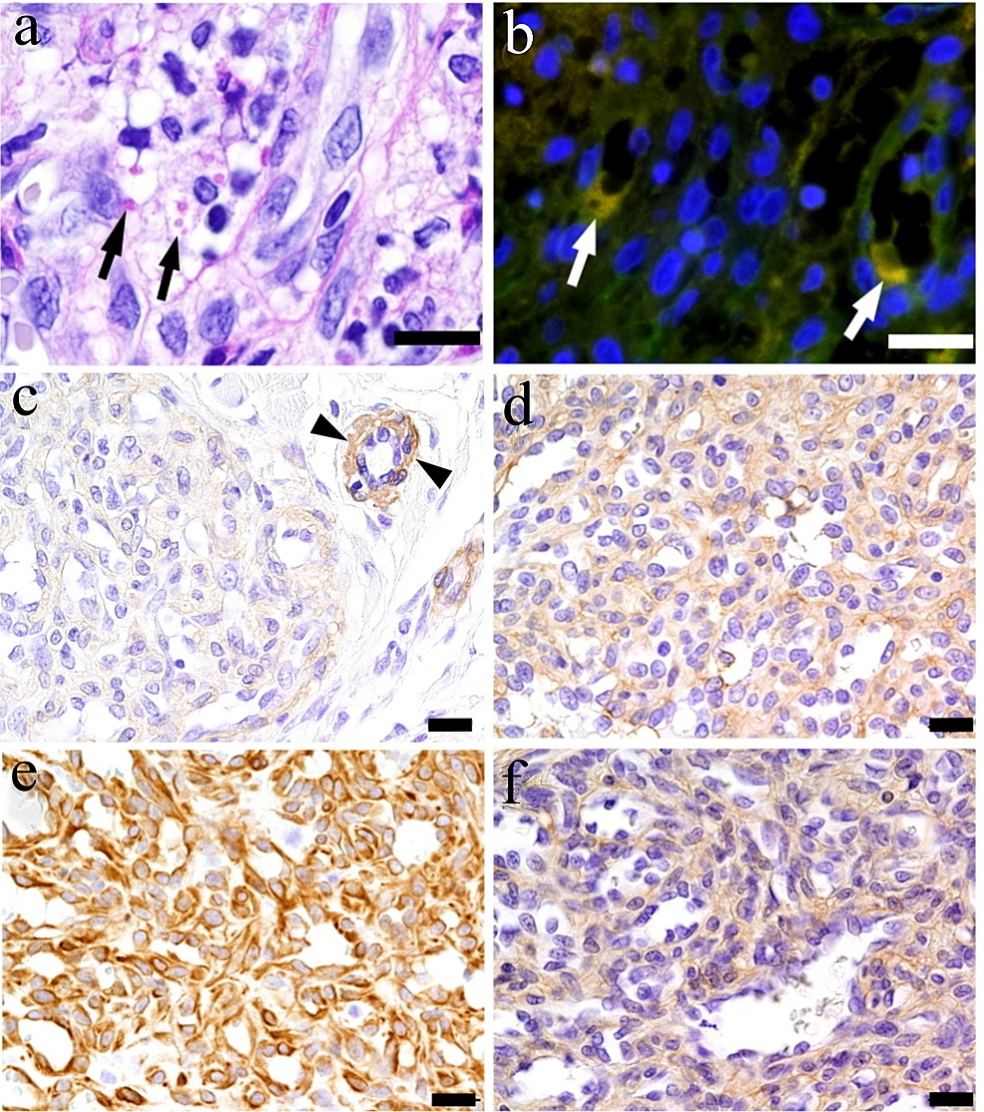
Histochemical and immunohistochemical features: (a) PAS staining highlights cytoplasmic granules (black arrows) in the cytoplasm; (b) Double immunofluorescence staining shows CD31-positive endothelial cells (green) and yellow cytoplasmic IgGκ deposition (white arrows); (c) αSMA is faintly positive in stromal cells but positive in normal vascular smooth muscle cells (arrowheads); Stromal cells are positive for (d) CD 146, (e) nestin, and (f) type IV-collagen Scale bars: 10 µm in (a) and (b), and 20 µm in (c)-(f) PAS: periodic acid-Schiff; αSMA; α-smooth muscle actin

Periodic acid-Schiff (PAS) staining highlighted intracytoplasmic globules of variable size (Figure 2a). Double immunofluorescence staining with CD31 (green) and IgGκ (red) showed that CD31-positive cells formed anastomosing cords that partially lined dilated lumens and lacked obvious vascular lumens in part. Some CD31-negative cells were intermingled between cords. The anastomosing cords were also positive for CD34 but not podoplanin (clone D2-40). Double-positive yellow reactions against CD31 and IgGκ antibodies confirmed immunoglobulin deposition in cytoplasm of CD31-positive endothelial cells (Figure 2b). Tumor cells were only faintly positive for α-smooth muscle actin (αSMA) compared with pericytes of normal vessels (Figure 2c). CD146 (Figure 2d) and nestin (Figure 2e) showed diffuse positivity in the tumor cells. Many tumor cells expressing CD146 and nestin, except for forming vascular lumens, were individually surrounded by type IV collagen-positive reaction (Figure 2f). A few CD68-positive histiocytic cells were found in the interstitial space. The patient showed no evidence of general symptoms and recurrence at the three-year follow-up after the surgery.

## Discussion

In 1990, Chan et al. first described the histological features of cutaneous glomeruloid hemangioma in two patients of multicentric Castleman Disease associated with POEMS syndrome [1]. They described three types of cells in their cases based on the expression patterns of factor VIII and muscle-specific actin: endothelial cells, pericyte, and immature stromal cells with endothelial nature. In this report, we describe the detailed histological and phenotypical characteristics of an intraoral GH without POEMS syndrome.

Although GH is thought to be a reactive lesion rather than a true neoplasm, malignant and other similar vascular lesions need to be ruled out. Lobular capillary hemangioma (pyogenic granuloma), acquired tufted hemangioma, papillary hemangioma, and Kaposi sarcoma should be considered as differential diagnoses. Lobular capillary hemangioma, rarely demonstrating an intravascular pattern, shows multiple lobules comprising capillaries with a reminiscence of the glomerulus. However, lobular capillary hemangioma is composed of endothelial cells surrounded by αSMA-positive-positive pericytes [7]. The presence of pericytes can easily discriminate lobular capillary hemangioma from GH. Acquired tufted hemangioma also demonstrates a lobular or cannonball pattern composed of small capillaries. However, this is histologically composed of cannonballs surrounded by podoplanin-positive lymphatic vessels. In addition, it macroscopically exhibits patch or plaque-like lesions rather than elevation [1]. Papillary hemangioma and Kaposi sarcoma demonstrate papillary proliferation and glomeruloid feature, respectively. The absence of spindle cell proliferation and slit-like vascular space can rule out Kaposi’s sarcoma [1]. Papillary hemangioma shows many similarities with GH, but morphology can be a clue to separate these two lesions. Capillaries of papillary hemangioma are lined by a single layer of normal-appearing endothelial cells with outer layers of pericytes in contrast to the predominant perivascular stromal cells of GH. Glomeruloid arrangement of capillary loops is never observed in papillary hemangioma [8,9].

GH without POEMS syndrome is challenging to diagnose; hence, it is essential to discuss the detailed histological characteristics. This case showed that multiple intravascular nodules with irregular vascular lumens lined by plump endothelial cells, and their interstitial spaces, were occupied by plump stromal cells as Chan et al. emphasized [1]. They also described that these stromal cells had endothelial nature in their case of GH. All of these structures collectively mimic capillary loops of renal glomeruli, which is a specific morphological feature for GH.

Immunophenotypically, glomeruloid nodules were composed of CD31-positive cords made up of plump stromal cells that were also positive for CD146 and nestin in this case. The CD31-positive stromal cells closely resemble factor VIII-positive stromal cells reported by Chan et al. [1]. An αSMA-positive-negative or faintly positive status might reflect the lack of mature pericytic nature. CD 146 is known as a pericytic marker, and CD146-positive and CD31-negative stromal cells act as progenitor cells for endothelial cells in angiogenesis [10]. Nestin, known as a neural stem/progenitor cell marker [11], was expressed in the populations of endothelial cells and immature stromal cells. Furthermore, these stromal cells were individually surrounded by type IV collagen, which is reminiscent of the Glomus cell, a specific type of pericyte. These findings suggest that the glomeruloid structure is occupied by immature pericytes capable of differentiating into endothelial cells, which seems to characterize this lesion. As mentioned above, this case is microscopically consistent with GH.

The present case did not show any symptoms such as polyneuropathy, monoclonal plasma cell proliferative disorder, Castleman disease, sclerotic bone lesions, and vascular endothelial growth factor elevation listed as major diagnostic criteria of POEMS syndrome [12] even three years after surgery. It is inevitable that the absence of these symptoms makes the final diagnosis of GH uncertain. However, GH can present for as long as 10 years before the full development of POEMS syndrome [5]. Considering the typical histological findings of this case, further careful follow-up is required.

## Conclusions

The presented case shows a characteristic morphology composed of many immature stromal cells occupying intercellular spaces of endothelial cells. This feature can differentiate GH from other vascular lesions with a glomeruloid appearance. Although no systemic disease was found in this patient to fulfill the criteria for POEMS syndrome, this case might be GH in the stage of slowly developing POEMS syndrome. Therefore, further careful follow-up will still be necessary.
